# Systematic Fusion of Multi-Source Cognitive Networks With Graph Learning - A Study on Fronto-Parietal Network

**DOI:** 10.3389/fnins.2022.866734

**Published:** 2022-07-29

**Authors:** Xiaofei Zhang, Yang Yang, Hongzhi Kuai, Jianhui Chen, Jiajin Huang, Peipeng Liang, Ning Zhong

**Affiliations:** ^1^Faculty of Information Technology, Beijing University of Technology, Beijing, China; ^2^School of Computer, Jiangsu University of Science and Technology, Zhenjiang, China; ^3^International WIC Institute, Beijing University of Technology, Beijing, China; ^4^Beijing International Collaboration Base on Brain Informatics and Wisdom Services, Beijing, China; ^5^Department of Psychology, Beijing Forest University, Beijing, China; ^6^Department of Life Science and Informatics, Maebashi Institute of Technology, Maebashi, Japan; ^7^School of Psychology and Beijing Key Laboratory of Learning and Cognition, Capital Normal University, Beijing, China

**Keywords:** systematic fusion, brain atlas, cognitive network, fronto-parietal network, fMRI

## Abstract

Cognitive tasks induce fluctuations in the functional connectivity between brain regions which constitute cognitive networks in the human brain. Although several cognitive networks have been identified, consensus still cannot be achieved on the precise borders and distribution of involved brain regions for each network, due to the multifarious use of diverse brain atlases in different studies. To address the problem, the current study proposed a novel approach to generate a fused cognitive network with the optimal performance in discriminating cognitive states by using graph learning, following the synthesization of one cognitive network defined by different brain atlases, and the construction of a hierarchical framework comprised of one main version and other supplementary versions of the specific cognitive network. As a result, the proposed method demonstrated better results compared with other machine learning methods for recognizing cognitive states, which was revealed by analyzing an fMRI dataset related to the mental arithmetic task. Our findings suggest that the fused cognitive network provides the potential to develop new mind decoding approaches.

## 1. Introduction

Cognitive functions of the human brain rely on neuronal activities, as well as the intra-neural networks and inter-neural networks. Modern neuroimaging technologies, such as functional magnetic resonance imaging (fMRI), have provided effective approaches to revealing the patterns of the neural network, also known as cognitive network, during the cognitive processes. Being a vital control-type cognitive network, the fronto-parietal network (FPN) occupying brain regions across the lateral prefrontal cortex to the posterior parietal cortex, plays a critical role in imposing cognitive control on a variety of tasks by initiating and deploying executive control abilities. As a result, it has always been in a flexible state full of dynamic changes while other processing-type cognitive networks are deemed to be more comparatively modular and static (Dosenbach et al., 2008). Previous studies on structural and functional neuroimaging have reached a general consensus that the FPN is responsible for intelligence, integrated with the cognitive functions including perception, attention, memory, language, and planning (Colom et al., 2010). Many fMRI and PET studies on attention, working memory, and episodic memory retrieval have reported the frequent detection of the FPN's activity. Moreover, the activation of the FPN was observed in some fMRI studies for conscious visual perception (Naghavi and Nyberg, 2005), and the FPN in the Theta band was found to take a vital role in a mentally demanding arithmetic task (Mizuhara and Yamaguchi, 2007). Consequently, the exploration of the FPN can help to provide a more comprehensive and precise understanding of the intelligence and cognitive abilities of the human brain.

Given that the human brain is a precisely interconnected system network, graph theory has increasingly proved to be a popular tool for the analysis of human MRI data (Fornito, 2016). By adopting graph analysis, it is found that the local and global integrity of the FPN, the cingulo-opercular network (CON), and other control-type cognitive networks, are significantly positively associated with cognitive abilities. It suggests that greater network efficiency supports better cognitive ability, evidenced by the similar performance in healthy participants and patients with schizophrenia (Sheffield et al., 2015). Under the resting state, the functional connectivity between the critical regions of the FPN is identified to be linked to the cognitive performance of patients with glioma as well as their cognitive outcome after the surgery treatment (Lang et al., 2017). The FPN and its subregions can change the functional connectivity with nodes of other cognitive networks on the different goals of cognitive tasks. In addition, the functional connectivity pattern of the FPN can indicate its involvement in specific tasks, and facilitate the novel tasks' learning in the form of a transferable code (Zanto and Gazzaley, 2013). Therefore, the FPN can be regarded as a defined control network, whose partial function is to interact with and change other cognitive networks (Marek and Dosenbach, 2018).

The cognitive networks in the human brain are often defined on the basis of anatomical or functional brain atlases. Automated Anatomical Labeling (AAL) is a commonly used anatomy-based structural brain atlas (Tzourio-Mazoyer et al., 2002). On the basis of AAL, the FPN and its default mode network (DMN) can be structurally defined, where the FPN has six regions in the frontal lobe and four regions in the parietal lobe (Oliver et al., 2019). The FPN can also be defined with some functional brain atlases. For instance, Dosenbach-160 (Dosenbach et al., 2010) is a human brain atlas consisting of 160 regions of interest (ROIs), where each ROI is uniquely assigned to one of its six cognitive networks. Power-264 (Power et al., 2011) is a human brain atlas composed of 264 ROIs, of which 236 ROIs are uniquely assigned to the given part of its 13 cognitive networks yet the remaining 28 ROIs belong to which part of the cognitive networks remains uncertain. There are 21 ROIs and 28 ROIs identified in the FPNs of Dosenbach-160 and Power-264, respectively. Willard-499 (Richiardi et al., 2015) is a voxel-defined brain atlas, and its 142 regions can be identified in one of its 14 cognitive networks while the cognitive networks to which the remaining 357 regions belong are unknown. Willard-499 does not make an explicit definition of the FPN, yet it defines two executive control networks (ECNs), namely, the left ECN and the right ECN located in the two hemispheres. Given that FPN and ECN are conceptually equivalent (Seeley et al., 2007; Vincent et al., 2008), the FPN can be obtained by tailoring the ECN of Willard-499. Moreover, some researchers defined a small brain atlas manually for their own research. For example, the brain atlas Gao-32 (Gao and Lin, 2012) consists of 32 ROIs, where each ROI is uniquely assigned to one of its five cognitive networks. The FPN of Gao-32 contains nine ROIs in the frontal lobe, parietal lobe, and insula. Despite the conceptual consistency in cognitive neuroscience, the above mentioned frameworks of FPN differ in their structures and distributions. The differences between the multiple FPNs bring challenges to exploring the processing mechanisms of FPNs in human brain cognitive tasks.

**Figure 1:**
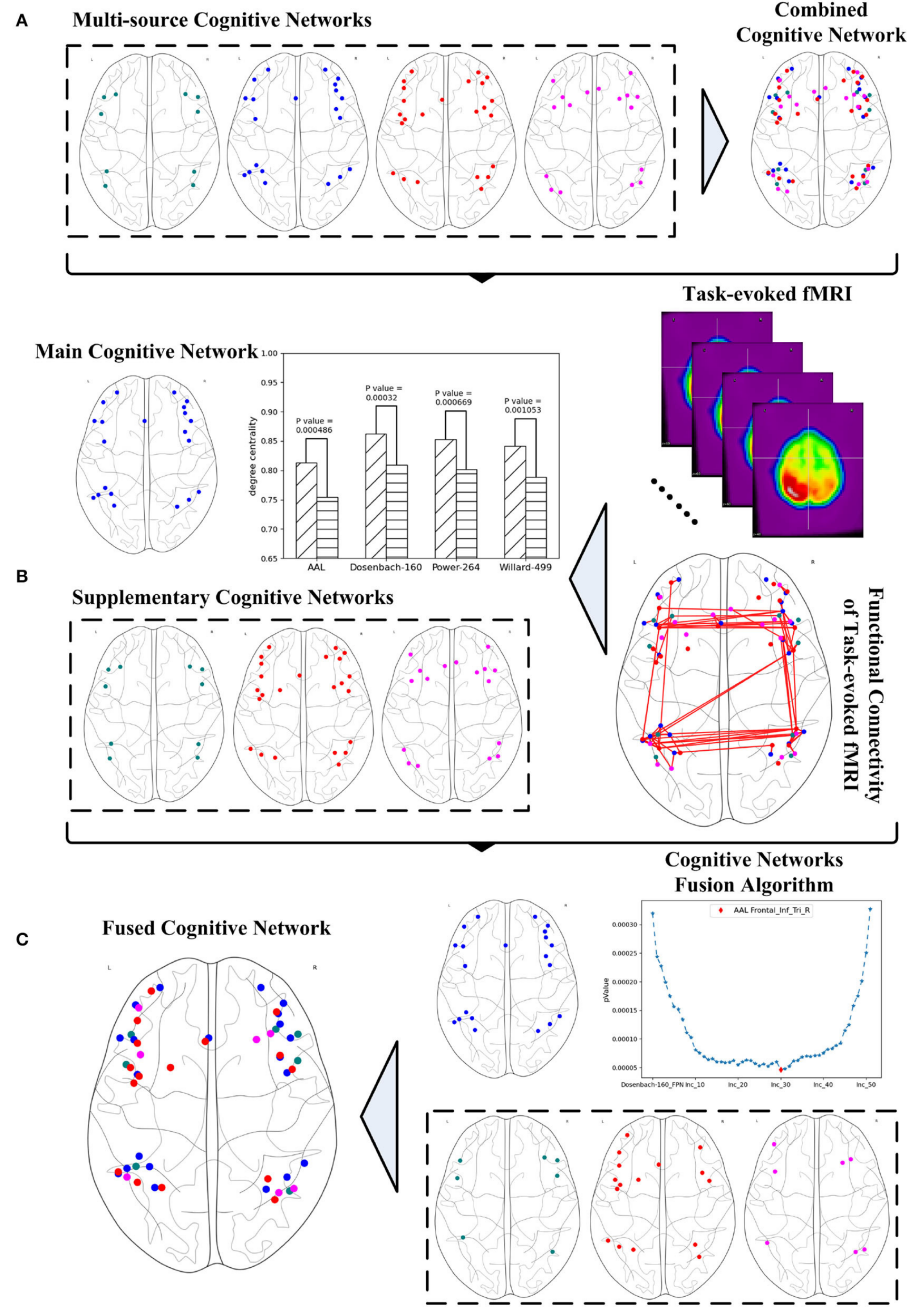
Systematic fusion of multi-source cognitive networks, **(A)** combined cognitive network synthesized from multiple brain atlases, **(B)** main and supplementary cognitive networks obtained from graph analysis, **(C)** fused cognitive network computed from cognitive networks fusion algorithm.

The various versions of the FPN definitions result in the fact that any exploration of the FPN from just a single perspective may only result in one sided outcome. Given the complexity of brain science, brain informatics (Zhong et al., 2011) claimed the importance and necessity of a thorough exploration for the research of human information processing system (HIPS). Thus, a systematic fusion of multi-source FPNs may provide an effective way to address this problem by providing a comprehensive and systematic investigation. This article aims to propose a graph-learning-based method for fusing multi-source cognitive networks and tends to evaluate it with its application to the fusion of the FPNs from multiple brain atlases. Three steps are involved to achieve this method as shown in Figure 1. The first step is to study and adjust the FPNs defined in the multiple brain atlases, so as to ensure the consistent boundaries between the regions contained in all the FPNs and to spatially synthesize the multi-source FPNs for the realization of a combined FPN. The second step is to analyze the functional connectivity of the combined FPN under a specific cognitive task and to calculate the graph properties of each independent FPN in the combined FPN. The single FPN with the optimal performance in discriminating the graph properties under different cognitive states is chosen as the main FPN while other FPNs are accepted as the supplementary FPNs. In the last step, the main cognitive network is adopted as the initial candidate fused FPN, into which the ROIs in all the supplementary FPNs are added one by one according to their nodal index. The iteratively fused FPN composed of the main FPN and the added ROIs has the optimal index and, thus, is set as the final fused FPN. The experiment materials are the fMRI data generated by mental arithmetic task (Yang et al., 2017) and the resulting fused FPN will be evaluated by comparing it with other classic machine learning methods.

## 2. Methodology

### 2.1. Synthesizing Multi-Source Cognitive Networks

Closely related to the core cognitive functions of the human brain, the FPN has been widely discussed in the research literature on brain atlases. However, no agreement has been reached on the definition of the FPN and its relative descriptions, and some obvious differences or even contradictions still exist between different brain atlases. To better explore the fusion of cognitive networks congruent with the FPN, this section will first analyze several typical brain atlases that define the FPN, and then clip some FPNs to obtain a reasonable combined FPN. Given the FPN is just one specific type of cognitive network in the human brain, it is necessary to generalize the definitions of cognitive network initially.

**Definition 1**. *CN* is the concept of a cognitive network that specializes in specific cognitive functions of the human brain, and (*CN*)^*I*^ refer to the set of all the concrete cognitive network instances of *CN*. *CN*_*inst*_, the element in the set of (*CN*)^*I*^, is one instance of *CN* and constituted by a collection of ROIs, as shown in Equation 1.

(1) $$\begin{array}{r} CN_{inst}=\left\{ROI_{1},ROI_{2},\ldots ,ROI_{\left|CN_{inst}\right|}\right\},CN_{inst}\in {\left(CN\right)}^{I} \end{array}$$

where |*CN*_*inst*_| is the ROIs' number of *CN*_*inst*_.

The ROIs in different instances of *CN* vary from different reference brain atlases. If a specific *CN* has the instances of $CN_{1},CN_{2},\ldots ,CN_{\left|{\left(CN\right)}^{I}\right|}$, the union of all the instances constitutes the combined cognitive network of *CN*, defined and shown in Equation 2.

**Definition 2**. A combined cognitive network noted as *CCN* and formulated in Equation 2 is the set of ROIs from all the instances of the same specific *CN*. |(*CN*)^*I*^| is the instances' number of the specific *CN*.

(2) $$\begin{array}{r} CCN=\overset{\left|{\left(CN\right)}^{I}\right|}{\underset{i=1}{⋃}}CN_{i},CN_{i}\in {\left(CN\right)}^{I} \end{array}$$

It should be noted that ROIs with equal Montreal Neurological Institute (MNI) coordinates may come from different instances of *CN*. Given that these ROIs originate from different brain atlases or research, the ROIs with the same MNI coordinates are still considered to be different. That is, $CN_{i}⋂CN_{j}=\emptyset$, $\forall CN_{i},\forall CN_{j}\in {\left(CN\right)}^{I},i\neq j$. With regards to the different ROIs from the same instance of *CN*, they do not have the same MNI coordinates.

**Figure 2:**
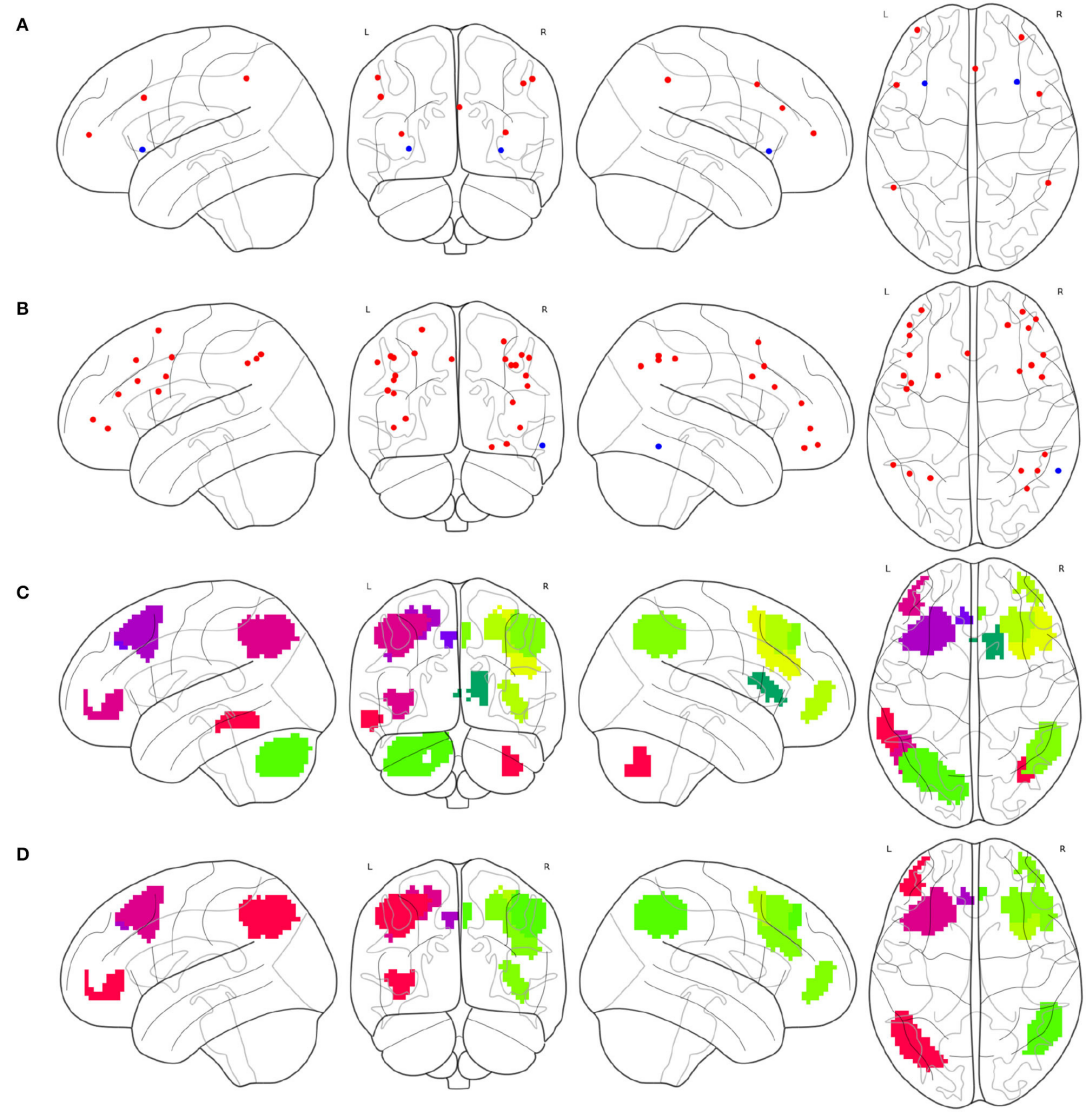
Definition issues of the FPNs in some brain atlases, **(A)** the ROIs in the FPN of Gao-32, where the blue ROIs belong to the insula, **(B)** the ROIs in the FPN of Power-264, where the only blue ROI belongs to the temporal lobe, **(C)** the regions in the ECN of Willard-499, where some of the regions belong to limbic lobe, temporal lobe, or cerebellum, and **(D)** the frontal and parietal regions in the ECN of Willard-499.

To synthesize a combined cognitive network with the FPNs as a specific case, the investigation of the ROIs contained in each instance of the FPN must be initially conducted. The brain atlas Gao-32 defines an FPN with nine ROIs, as shown in Figure 2A, of which seven red ROIs belong to the frontal lobe or parietal lobe, while the two blue ROIs belong to the insula. The present study does not define the insula as the portion of the FPN. Previous emotion-controlling studies on the exploration of the emotion-regulating strategies failed to prove the possible connections between insula-active and FPN-active regions (Li et al., 2021), which indicates that the insula may not be a part of the FPN on the edge. Suppose these results are correct, there are only seven ROIs left in the FPN of Gao-32 after the removal of the insula ROIs. Moreover, very scant follow-up studies were conducted to make a further validation of Gao-32, thus the FPN of Gao-32 is not on the consideration list for the present study. The brain atlas Power-264 defines an FPN with 25 ROIs, as shown in Figure 2B, in which 24 red ROIs belong to the frontal lobe or parietal lobe, while the only blue ROI belongs to the temporal lobe. Although previous studies have indicated that many cognitive networks vary among subjects, a great level of overlap can be identified from the FPN of multiple subjects in the internal parietal sulcus, ventral inferior temporal gyrus, and lateral prefrontal cortex (Marek and Dosenbach, 2018). Given that one ROI in the FPN of Power-264 belongs to the middle temporal gyrus in the temporal lobe, it is removed from the definition of the FPN with the Power-264 in the present study. The brain atlas Willard-499 defines an ECN containing 24 regions, as shown in Figure 2C, 17 of which belong to the frontal lobe or parietal lobe. In addition, there is one specific region in both limbic and temporal lobes and five particular regions in the cerebellum. Considering Willard-499 is defined with numbers for different regions rather than with specific names, only the regions located in the frontal lobe or parietal lobe in the ECN of Willard-499 are retained, so as to reach the congruity between the Power-264 clipping strategy and the lobes involved in the FPN of other brain atlases. A customized Willard-499 FPN is finally obtained as shown in Figure 2D.

**Figure 3:**
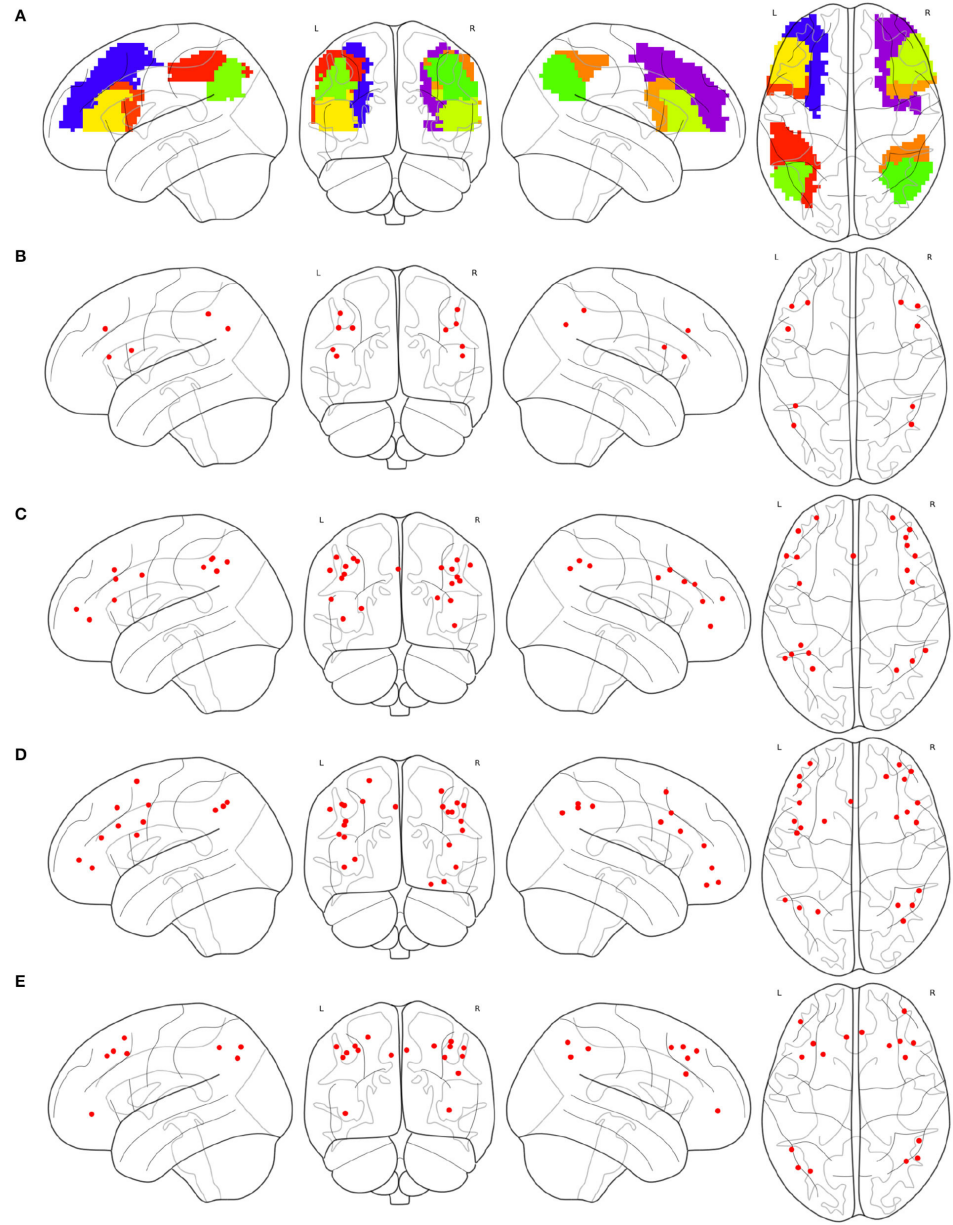
Multi-source FPNs for synthesizing the combined FPN, **(A)** the regions belong to the FPN of AAL, **(B)** 10 ROIs of the FPN in AAL, **(C)** 21 ROIs of the FPN in Dosenbach-160, **(D)** 24 ROIs of the FPN in Power-264, and **(E)** 17 ROIs of the FPN in Willard-499.

Partial brain atlases defined their FPNs containing regions or ROIs that only belong to the frontal lobe or parietal lobe, resulting in the congruity between conceptual boundaries when synthesizing multi-source FPNs. The FPN defined on the basis of AAL contains 10 regions (Oliver et al., 2019), all of which belong to the frontal lobe or parietal lobe, as shown in Figure 3A. The FPN obtained from the central ROI of each region is shown in Figure 3B. Dosenbach-160 defines an FPN with 21 ROIs, and all ROIs belongs to the frontal lobe or parietal lobe exclusively, as shown in Figure 3C. The original FPN of Power-264 is clipped to obtain a pruned FPN with 24 ROIs, as shown in Figure 3D. An FPN with 17 regions is obtained by clipping the original Willard-499 ECN, and the ROIs in the new FPN of Willard-499 are shown in Figure 3E.

In the present study, the original or clipped FPNs with reference to AAL, Dosenbach-160, Power-264, and Willard-499 are confined to the frontal lobe and parietal lobe, providing the congruent lobe boundaries to synthesize multi-source FPNs. The combined FPN contains 72 ROIs, located in the frontal lobe or parietal lobe, and their distributions in the brain are shown in Table 1. Of all the forty-seven frontal ROIs in the combined FPN, six come from AAL, 13 from Dosenbach-160, 17 from Power-264, and 11 from Willard-499. On top of that, four ROIs from AAL, eight ROIs from Dosenbach-160, seven ROIs from Power-264, and six ROIs from Willard-499 constitute the 25 parietal ROIs in the combined FPN.

**Table 1:** Combined fronto-parietal network.

**Cognitive network**	**Number of ROIs**					
	**Frontal**	**Parietal**	**Occipital**	**Temporal**	**Limbic**	**Cerebellum**
FPN of AAL	6	4	0	0	0	0
FPN of Dosenbach-160	13	8	0	0	0	0
FPN of Power-264	17	7	0	1	0	0
ECN of Willard-499	11	6	0	1	1	5
Combined FPN	47	25	0	0	0	0

Taking the *FPN* as a specific concept of the cognitive network as an example, if the four FPNs mentioned above make up all the instances of the *FPN*, the definition of the instances set of the *FPN* can be shown in Equation 3.

(3) $$\begin{array}{r} {\left(FPN\right)}^{I}=\left\{FPN_{a},FPN_{d},FPN_{p},FPN_{w}\right\} \end{array}$$

where *FPN*_*a*_, *FPN*_*d*_, *FPN*_*p*_, and *FPN*_*w*_ represent the FPNs in the brain atlas of AAL, Dosenbach-160, Power-264, and Willard-499, respectively.

To arrive at a comprehensive examination of all the FPNs defined in these human brain atlases, all the contained ROIs need to be synthesized. And the definition of the combined FPN is shown in Equation 4.

(4) $$\begin{array}{r} CombinedFPN=FPN_{a}⋃FPN_{d}⋃FPN_{p}⋃FPN_{w} \end{array}$$

Compared with other FPN instances, more ROIs in *CombinedFPN* mean more various dimensions of the correlation matrices for functional connectivity analysis, resulting in the difficulty in interpreting the final results of the *CombinedFPN*. Consequently, the following section will discuss how to set a given FPN instance as the main FPN in the *CombinedFPN*, and then choose some ROIs with higher priority from the remaining supplementary FPNs to construct a fused FPN so as to ensure a reasonable range of the dimensions, as well as the optimal performance in discriminating the graph properties of the FPN under different cognitive states.

### 2.2. Selecting Main Cognitive Network

Although the synthesis of multi-source cognitive networks can examine more cortical regions relevant to the cognitive task, such an operation will contribute to the increase of computational load during data analysis, as well as the worse performances in discriminating the graph properties of the FPN under different cognitive states. Alternatively, the concentration on one specific instance of the cognitive network may help to provide a consensus to reach a better interpretation of the analyzed results. Consequently, there is a need to select one instance of *CN* as the main cognitive network and set the remaining instances of *CN* as the supplementary cognitive networks.

**Definition 3**. The main cognitive network noted as *MCN* and formulated in Equation 5 is the instance of *CN* and has the optimal performance, compared with any other instances of *CN*, in discriminating the graph properties under different cognitive states for the relative task.


(5) $$\begin{array}{r} MCN\in {\left(CN\right)}^{I},P\left(MCN\right)\geq P\left(CN_{i}\right),CN_{i}\in {\left(CN\right)}^{I},CN_{i}\neq MCN \end{array}$$

where *P* stands for the performance, usually set as the *P*-value, in discriminating the graph properties of the FPN under different cognitive states. The *P*-*Value* of each instance of *CN* is given in Algorithm 1. The remaining instances of *CN* excluding *MCN* are the supplementary cognitive networks.

**Algorithm 1:**
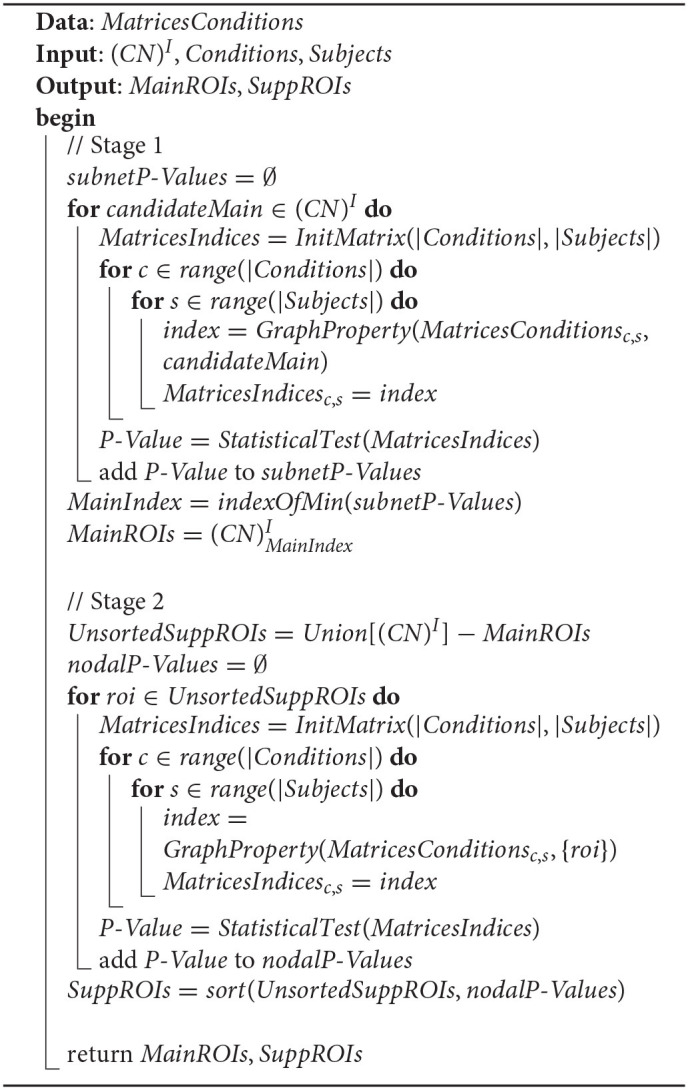
Main cognitive network selection.

**Definition 4**. The supplementary cognitive network noted as *SCN* and formulated in Equation 6 refers to any instances of *CN* excluding *MCN*.


(6) $$\begin{array}{r} SCN\in {\left(CN\right)}^{I}-\left\{MCN\right\} \end{array}$$

The selection procedure of *MCN* and *SCN* is depicted in Algorithm 1, with the manipulated data *MatricesConditions*. The input parameters include (*CN*)^*I*^, *Conditions*, and *Subjects*, and the output parameters include *MainROIs* and *SuppROIs*. (*CN*)^*I*^ is the set of all instances of *CN*, while *Conditions* and *Subjects* are arrays containing the conditions and subjects of the cognitive task respectively. The returned *MainROIs* are a non-priority list of ROIs from *MCN*, and *SuppROIs* are a prior list of ROIs from all *SCNs*. Algorithm 1 can be divided into two stages as follows:

**Stage 1**: Select the cognitive network in (*CN*)^*I*^ with the optimal performance as the *MCN*, and append the ROIs of *MCN* into a non-priority list as *MainROIs*.

**Stage 2**: Merge the ROIs of all *SCNs* in (*CN*)^*I*^ into a non-priority list as *UnsortedSuppROIs*, and reorder the ROIs in *UnsortedSuppROIs* into a priority list as *SuppROIs* according to their nodal graph property.

The data *MatricesConditions* is a four-dimensional matrix, where the first dimension refers to the conditions of the cognitive task, and the dimension size stands for the number of conditions. For instance, if the mental arithmetic task only involve the conditions of addition and subtraction, the dimension size will be set as two. The subjects recruited for the cognitive task stand for the second dimension, thus this dimension size is measured by the number of subjects. In the present study, twenty-one subjects participated in the present mental arithmetic task, and thus, the subjects' dimension size is 21. The third and fourth dimensions of the matrix both represent the nodes contained in the CCN. Since the combined FPN here contains a total of 72 nodes, the sizes of the two dimensions are both 72 in the current case.

**Figure 4:**
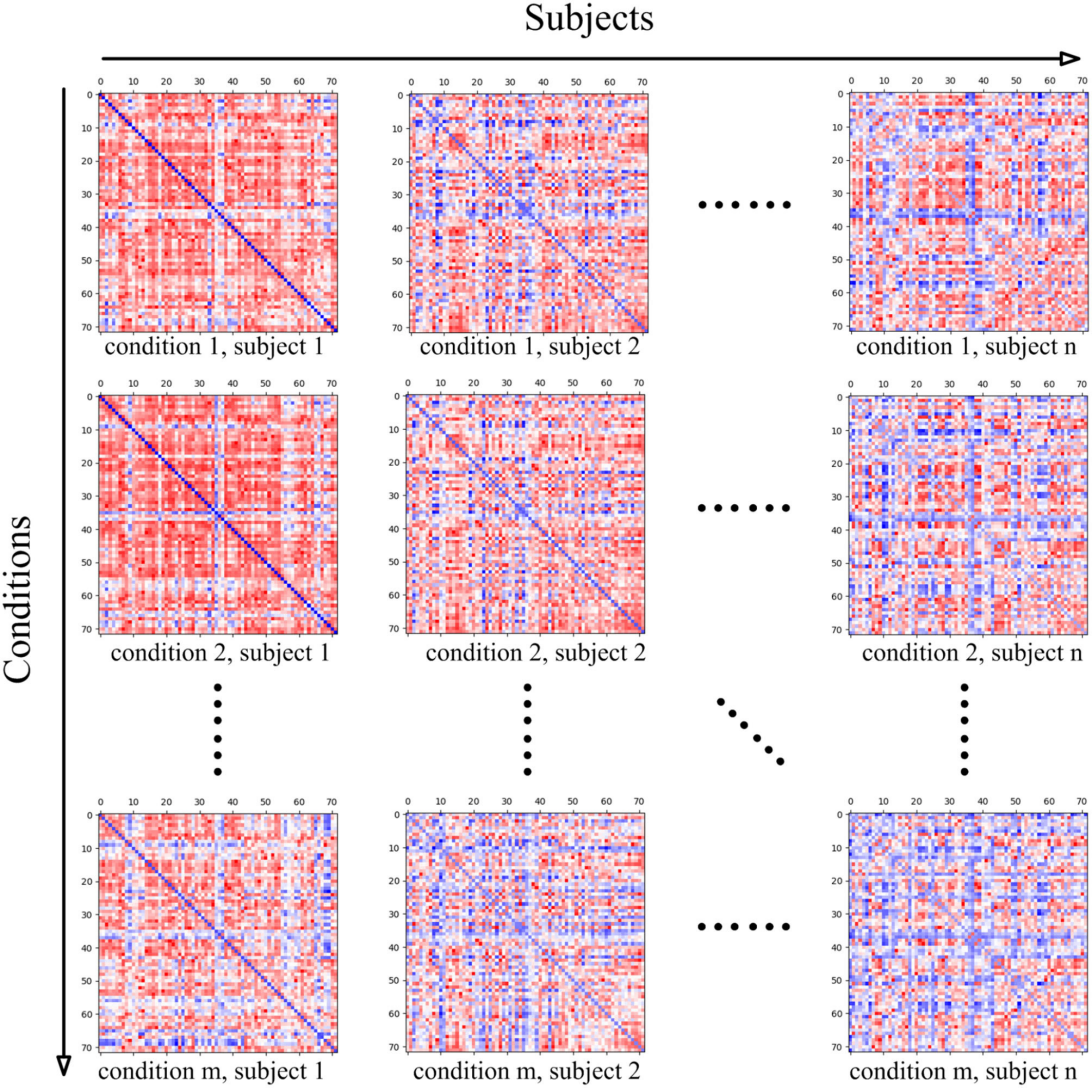
The four-dimensional matrix *MatricesConditions*, where the first dimension represents the conditions in the cognitive task, and the second dimension represents the subject. When the condition and subject are set, the obtained two-dimensional matrix stands for the brain functional connectivity matrix of the subject under the current cognitive task condition, and the shape of the two-dimensional matrix is determined by the ROIs' number of the adopted cognitive network.

The intermediate two-dimensional matrix *MatricesConditions*_*c,s*_ at both stages is the sub-matrix of *MatricesConditions* where *c* represents the index of the cognitive task condition in *Conditions* and *s* represents the index of the subject in *Subjects*. *MatricesConditions*_*c,s*_ basically stands for the functional connectivity matrix of the subject *Subjects*_*s*_ under the cognitive task condition *Conditions*_*c*_. Since the functional connectivity matrix of *MatricesConditions*_*c,s*_ is constructed on the basis of *CCN*, the shape of *MatricesConditions*_*c,s*_ is |*CCN*| × |*CCN*|. The relationship between *MatricesConditions* and its sub-matrix *MatricesConditions*_*c,s*_ is shown in Figure 4.

The functional connectivity matrix of *MatricesConditions*_*c,s*_ for each subject under specific cognitive conditions has been given, yet what actually needs to be calculated is the graph property of the candidate's main cognitive network, noted as *candidateMain* in the top loop at Stage 1, as well as the nodal graph property of the ROI, noted as *roi* in the top loop at Stage 2. Since *candidateMain* is a proper subset of *CCN*, and *roi* is an element of *CCN*, the graph property calculation implemented by the function of *GraphProperty* is based on *MatricesConditions*_*c,s*_ as the first parameter. The second parameter of *GraphProperty* is of great significance in that its setting size can determine whether the calculating processing is targeted at graph property or nodal graph property. The metric choice of graph property includes degree centrality, clustering coefficient, and network efficiency. In the present study, degree centrality is set as the metric for its popular application in discriminating the graph properties under different cognitive states.

The intermediate two-dimensional matrix *MatricesIndices* at the two stages is used for storing the performance of each candidate's main cognitive network in (*CN*)^*I*^ or the performance of each ROI in *UnsortedSuppROIs*. The shape of *MatricesIndices* is |*Conditions*| × |*Subjects*|, and *MatricesIndices*_*c,s*_ is the cell in the *cth* row and the *sth* column to store the performance corresponding to subject *Subjects*_*s*_ under cognitive condition *Conditions*_*c*_.

The function *StatisticalTest* at the two stages is used to conduct the statistical test between the rows of *MatricesIndices*, and return the *P*-*Value* which represents the performance in discriminating the graph properties under different cognitive states. The row number, namely the number of cognitive conditions, of *MatricesIndices* needs to be considered in the choice of a specific test function. If the row number of *MatricesIndices* is two, the statistical analysis of the *t*-test or the χ^2^ test can be adopted, yet if the row number is greater than two, the statistical analysis of variance needs to be utilized.

When the *P*-*Value* of each *candidateMain* at Stage 1 is obtained, the cognitive network with the minimum *P*-*Value* is selected as the main cognitive network, and the ROIs in the main cognitive network are appended into the non-priority list *MainROIs*. Similarly, after the acquisition of *P*-*Value* of each *roi* from *UnsortedSuppROIs* at Stage 2, the ROIs in *UnsortedSuppROIs* are reordered and appended into the prior list *SuppROIs*. Finally, the *MainROIs* and *SuppROIs* are returned by the algorithm.

### 2.3. Searching a Fused Cognitive Network

When *MCN*, consisting of the ROIs in *MainROIs*, is selected and set as the main cognitive network, it will be utilized as the base for fusing the ROIs, which are stored in the priority list *SuppROIs*, from all the supplementary cognitive networks.

**Definition 5**. Fused cognitive network, noted as *FCN* and formulated in Equation 7, is the union of *MCN* and the set, noted as *Sub*(*SuppROIs, fusedIndex*), constituted by the first number of *fusedIndex* ROIs in *SuppROIs*, and has the optimal performance in discriminating the graph properties under different cognitive states for the relative task.

(7) $$\begin{array}{r} FCN=MCN⋃Sub\left(SuppROIs,fusedIndex\right) \end{array}$$

The second parameter in *Sub* operation determines how many ROIs are chosen from the beginning of *SuppROIs* to constitute the set, and its value ranges from 0 to |*SuppROIs*|. *Sub*(*SuppROIs*, 0) means an empty set, and *Sub*(*SuppROIs*, |*SuppROIs*|) means the set containing all the ROIs in *SuppROIs*. Since *FCN* has the optimal performance, it can be inferred that *P*(*MCN* ⋃ *Sub*(*SuppROIs, fusedIndex*)) ≥ *P*(*MCN* ⋃ *Sub*(*SuppROIs, i*)), where *P* is the same as the one defined in Equation 5, and *i* ≠ *fusedIndex*.

Therefore, the essence of searching the *FCN* is to find the value of *fusedIndex* and to integrate the ROIs in *Sub*(*SuppROIs, fusedIndex*) into *MCN* to construct the *FCN*. The search process of the *fusedIndex* is implemented by Algorithm 2, which can be divided into two stages as follows:

**Stage 1**: Initialize the candidate fused cognitive network, represented as the list *iterFusedROIs*, with the ROIs in the non-priority list *MainROIs*. Then, add the ROIs in *SuppROIs* into the candidate fused cognitive network iteratively and append the performance of the current candidate fused cognitive network into the list of *P*-*Values*.

**Stage 2**: Select the minimum *P*-*Value* in *P*-*Values* and its corresponding position, namely *FusedIndex*, in the list. The ROI at the position of *FusedIndex* and all its leading ROIs in *SuppROIs*, together with the ROIs in *MainROIs*, constitute the final fused cognitive network *FCN* with the optimal performance in discriminating the graph properties under different cognitive states.

**Algorithm 2:**
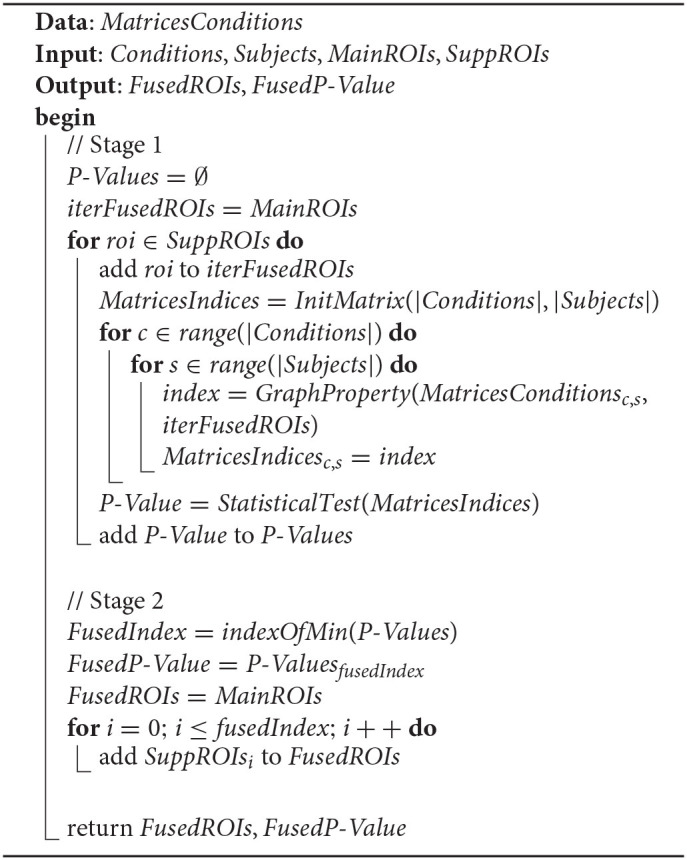
Cognitive networks fusion.

*MatricesConditions*, *Conditions*, and *Subjects* are the same as the ones in Algorithm 1 and will be reused. The returned *MainROIs* and *SuppROIs* by Algorithm 1 are set as the input parameters here. The output results, namely *FusedROIs* and *FusedP*-*Value*, are the generated list of ROIs in the optimal fused cognitive network *FCN* and its performance, respectively.

Despite the optimal performance in the entire iterative searching process, further comparisons between the fused cognitive network and the results obtained from other typical machine learning methods are necessity.

## 3. Experiments and Results

### 3.1. Experiment

The fMRI data comes from the mental arithmetic task of simple addition and subtraction (Yang et al., 2017) designed by the Web Intelligence Consortium (WIC). The goal of this cognitive task is to study the regularity of brain neural activity in simple arithmetic operations. Twenty-one subjects (12 males, 9 females) with no statistically significant differences were recruited. Before the experiment, each subject was made clear about the possible natural responses during the task process, and all subjects signed an informed consent form. After obtaining permission from the Ethics Committee of Xuanwu Hospital of Capital Medical University, the cognitive task was implemented and the fMRI data were collected by the WIC team in the hospital.

The preprocessing of the fMRI data was conducted with the software of Statistical Parameter Mapping (Friston et al., 2007) in four steps, namely slice time correction, head motion correction, spatial normalization, and smoothing. The Python software package and other relative software packages were used for the graph analysis of the fMRI data and the implementation of the cognitive networks fusion algorithm. The NiBabel was used for the basic manipulation of neuroimaging files like fMRI data and the SciPy (Virtanen et al., 2020) was used for Pearson coefficient calculation, FDR correction, and statistical test. Topological indices in the graph analysis were calculated by NetworkX (Hagberg et al., 2008), and the results were visualized *via* NiLearn.

### 3.2. Results

**Figure 5:**
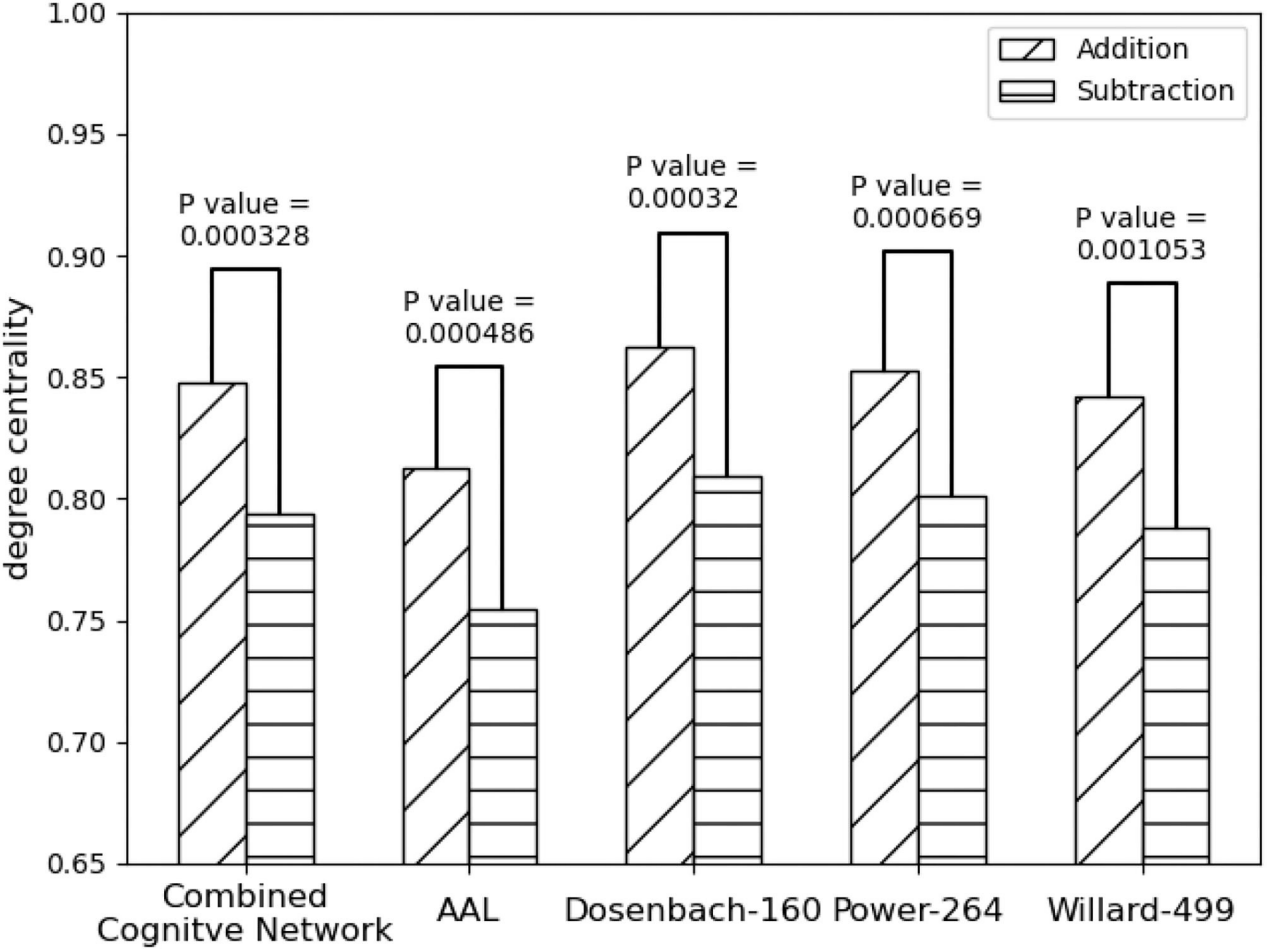
Statistical comparison results of the FPNs' topological property (degree centrality) between the addition and subtraction cognitive states. The FPN of Dosenbach-160 has yielded the sharp differences with the biggest statistical significance between the two cognitive states of mental arithmetic, followed by the FPNs of CCN (Combined Cognitive Network), AAL, Power-264, and Willard-499.

As shown in Figure 5 all the instances of *FPN*, *FPN*_*d*_ has the optimal performance of the statistical test (*P*-*Value* = 0.00032) between the degree centralities of the functional connectivity matrices under the two mental arithmetic cognitive states, followed by *CombinedFPN* (*P*-*Value* = 0.000328), *FPN*_*a*_ (*P*-*Value* = 0.000486), *FPN*_*p*_ (*P*-*Value* = 0.000669), and *FPN*_*w*_ (*P*-*Value* = 0.001053), respectively. Consequently, *FPN*_*d*_ is set as *MCN*, and the remaining instances of *FPN* are used as *SCN* in the following fusion of cognitive networks.

**Figure 6:**
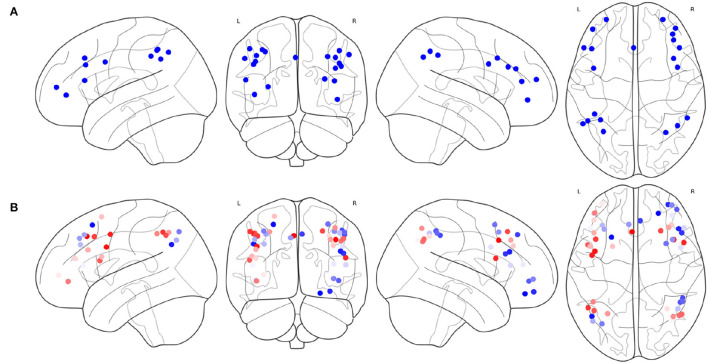
Results of the main FPN and supplementary FPNs, **(A)** the ROIs in *MainROIs*, i.e., the FPN in Dosenbach-160, **(B)** the ROIs in all the supplementary FPNs, i.e., the FPNs in AAL, Power-264, and Willard-499. ROI with warmer colors has a higher priority in *SuppROIs*. Conversely, ROI with cooler color has a lower priority in *SuppROIs*.

The result of Algorithm 1 is shown in Figure 6. *FPN*_*d*_ is selected as the main FPN and all the ROIs in *FPN*_*d*_ are in the returned non-priority list *MainROIs* as in Figure 6A. *FPN*_*a*_, *FPN*_*p*_, and *FPN*_*w*_ are all selected as the supplementary FPNs, all the ROIs are in the returned priority list *SuppROIs*. As shown in Figure 6B, the ROI with warmer color has a higher priority and will be in closer propinquity to the head of *SuppROIs*, while the ROI with cooler color has a lower priority and will be in closer propinquity to the tail of *SuppROIs*. The position of the ROI in *SuppROIs* determines the time when it will be added into the candidate FPN in the iterative process of Algorithm 2.

**Figure 7:**
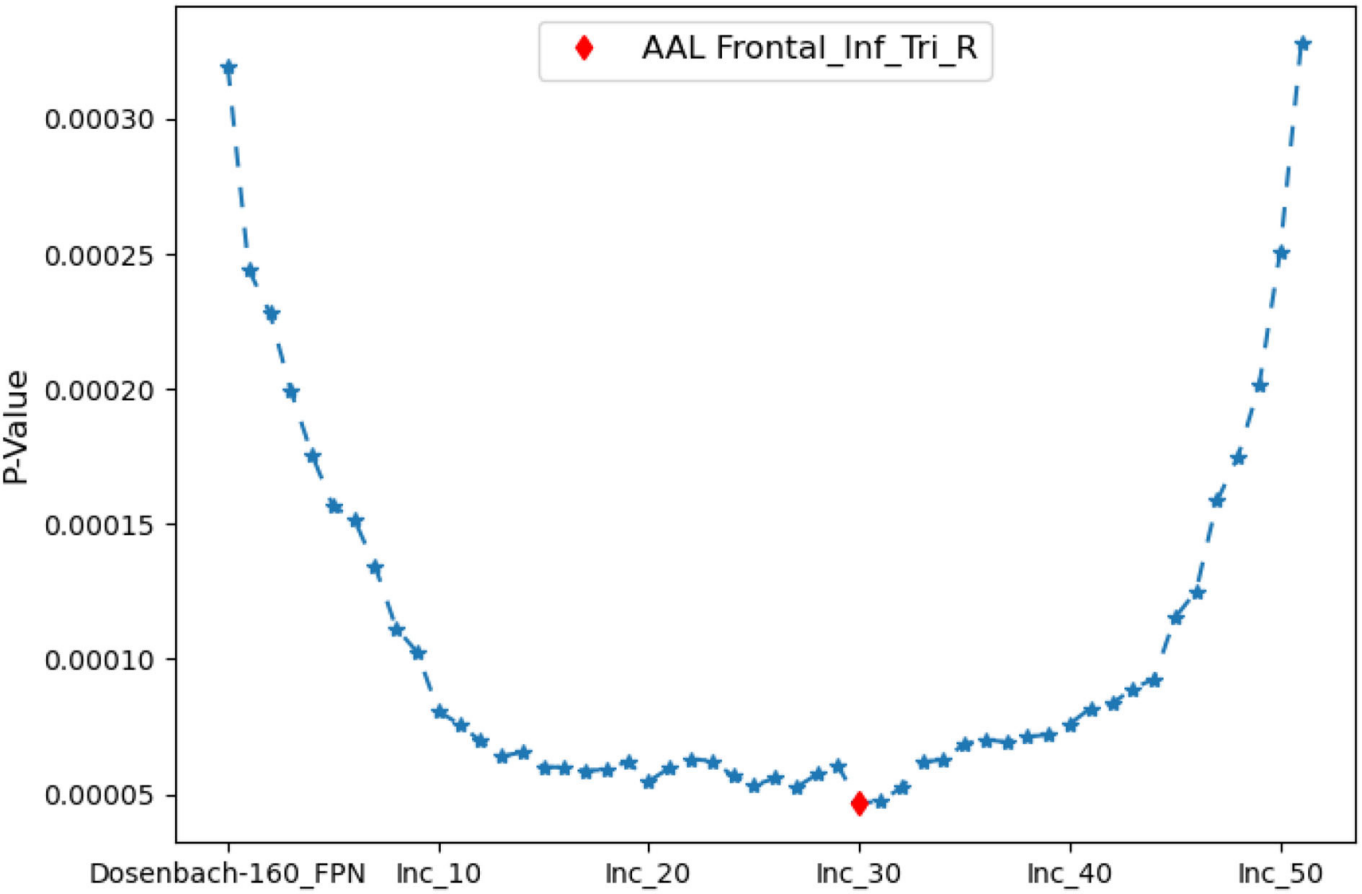
The iterative statistical test of the graph properties of the candidate fused FPNs in discriminating the cognitive states. When Dosenbach-160 is set as the initial candidate fused FPN, and the top thirty of all ROIs in AAL, Power-264, and Willard-499 are added to Dosenbach-160, the candidate fused FPN can produce the most significant statistical performance. The last ROI to be added to the fused FPN is the triangular part of the inferior frontal gyrus from the right hemisphere of AAL.

The iterative process of Algorithm 2 is displayed in Figure 7. *FPN*_*d*_ is used as the initial candidate fused FPN, and the ROIs in *SuppROIs* are added into the candidate fused FPN one by one. The performance of the candidate fused FPN in discriminating the graph properties under the two mental arithmetic conditions is statistically tested during each iteration. The results show that the candidate fused FPN, formed by the ROIs of *FPN*_*d*_ and the first 30 ROIs in *SuppROIs*, has the optimal performance. The triangular part of the inferior frontal gyrus from the right hemisphere of AAL is eventually added into the resulting fused FPN with 51 ROIs. As shown in Table 2, the numbers of ROIs belonging to the frontal and parietal lobes in the fused FPN are 33 and 18, respectively. More specifically, among the 33 ROIs in the frontal lobe, the numbers from the FPNs in brain atlas of AAL, Dosenbach-160, Power-264, and Willard-499 are 5, 13, 11, and and 4 respectively while the corresponding numbers are 2, 8, 5, and 3 in the parietal lobe respectively.

**Table 2:** Fused fronto-parietal network.

**Cognitive network**	**Number of ROIs**	
	**Frontal**	**Parietal**
FPN of AAL	5	2
FPN of Dosenbach-160	13	8
FPN of Power-264	11	5
ECN of Willard-499	4	3
Fused FPN	33	18

**Figure 8:**
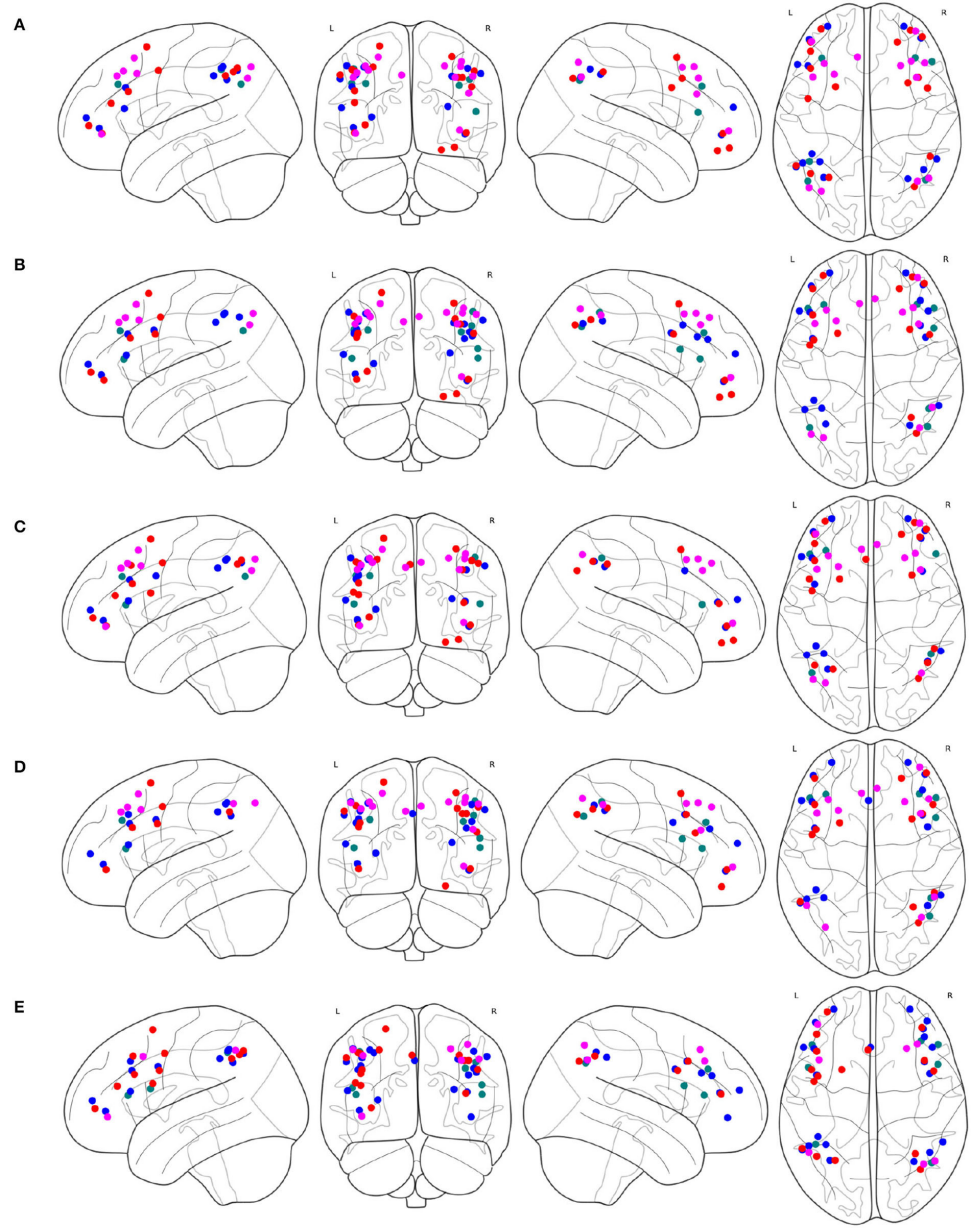
FPNs with 51 ROIs calculated by different methods. ROIs with colors teal, blue, red, magenta come from AAL, Dosenbach-160, Power-264, and Willard-499, respectively, **(A)** 6, 14, 17, and 14 ROIs are selected by ExtraTrees from the four brain atlases, respectively, **(B)** 8, 17, 13, and 13 ROIs are selected by AdaBoost from the four brain atlases, respectively, **(C)** 5, 15, 18, and 13 ROIs are selected by RandomForest from the four brain atlases, respectively, **(D)** 7, 17, 14, and 13 ROIs are selected by XGB from the four brain atlases, respectively, **(E)** 7, 21, 16, and 7 ROIs are selected by the cognitive networks fusion algorithm from the 4 brain atlases, respectively.

The spacial differences can be intuitively identified between the distributions of the ROIs of the generated FPNs with a close eye on Figure 8. Figures 8A–D display the FPNs with the top 51 ROIs chosen by ExtraTrees, AdaBoost, RandomForest, and XGB, respectively. The fused FPN calculated by Algorithm 2 is shown in Figure 8E. The numbers of ROIs located in the frontal lobe and parietal lobe are 31/20 (ExtraTrees), 36/15 (AdaBoost), 35/16 (RandomForest), 35/16 (XGB), and 33/18 (Algorithm 2). All these algorithms are conducted on the basis of *CombinedFPN*. Since *CombinedFPN* yields 47 ROIs and 25 ROIs in the frontal lobe and parietal lobe, respectively, it could be judged that the distribution ratio of ROIs in the two lobes generated from Algorithm 2 is close to that of *CombinedFPN*.

**Figure 9:**
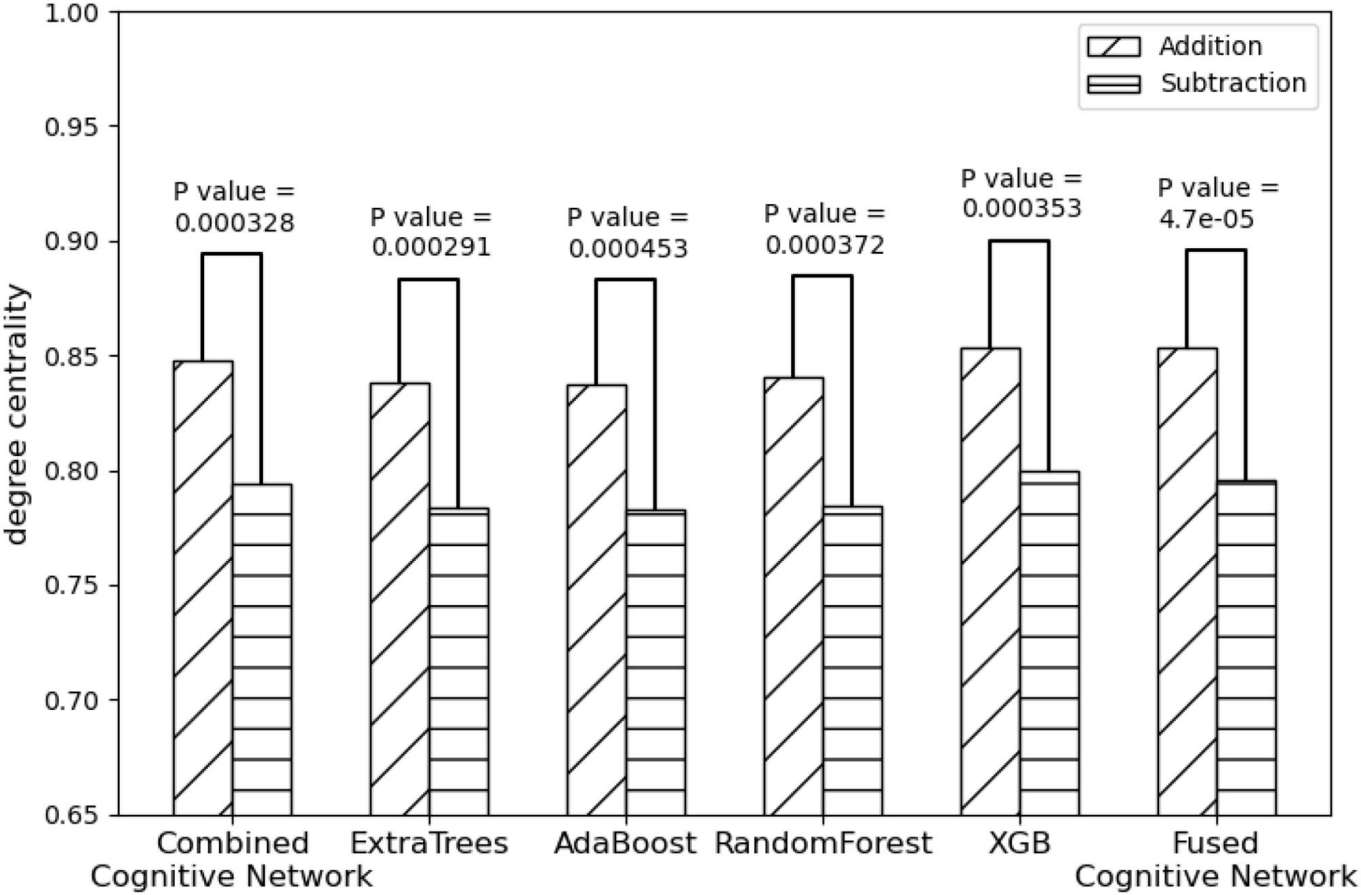
Statistical comparison results of the FPNs' topological properties between the addition cognitive state and the subtraction cognitive state. Among them, the fused FPN by the cognitive networks fusion algorithm has yielded the sharp differences with the biggest statistical significance between the two cognitive states of mental arithmetic, followed by the FPNs generated by ExtraTrees, CCN (Combined Cognitive Network), XGB, RandomForest, and AdaBoost.

On the other hand, compared with the adopted machine learning algorithms, Algorithm 2 has the optimal performance, as shown in Figure 9 and Table 3, of the statistical test (*P*-*Value* = 4.7*e*-05) between the degree centralities of the functional connectivity matrices under the two mental arithmetic cognitive states, followed by that of ExtraTrees (*P*-*Value* = 0.000291), XGB (*P*-*Value* = 0.000353), RandomForest (*P*-*Value* = 0.000372), and AdaBoost (*P*-*Value* = 0.000453). Such a performance is even better than that of *CombinedFPN* (*P*-*Value* = 0.000328). In a word, it can be safely concluded that Algorithm 2 can choose the FPN better representing the cognitive states of mental arithmetic.

**Table 3:** Performance of each method in discriminating cognitive states.

**Method**	**Mean of degree centrality**		***P*-Value**
	**Addition**	**Subtraction**	
Combined Cognitive Network	0.8474	0.7941	0.000328
ExtraTress	0.8384	0.7833	0.000291
AdaBoost	0.8371	0.7830	0.000453
RandomForest	0.8401	0.7845	0.000372
XGB	0.8536	0.7999	0.000353
Fused Cogntive Network	0.8536	0.7957	0.000047

## 4. Discussion

In the present study, the graph properties of the multi-source FPNs, combined FPN, fused FPN, and the FPNs generated by the adopted machine learning methods can be effectively used to discriminate the graph properties under different cognitive states in mental arithmetic task. Such a result lends support to the previous studies about the major dependence of adults' arithmetic ability on the FPN. To date, many research methods have been adopted to explore the possible role of the FPN in mental arithmetic, such as the meta-analysis of the brain regions involved in numbers and mental arithmetic (Arsalidou and Taylor, 2011), the pathway analysis on the brain mental arithmetic (Dehaenea and Cohen, 1997), and the structural connection analysis on the code model involved in mental arithmetic (Klein et al., 2013, 2016). All these studies proved that the mental arithmetic processing can activate the adult brain's FPN, which consists of the superior parietal lobule (SPL) and inferior parietal lobule (IPL) in the parietal regions, and inferior frontal gyrus (IFG), middle frontal gyrus (MFG), and left superior frontal in frontal regions.

The FPN plays an important part in adults' mental arithmetic processing, and a similar network was also detected in children's mental arithmetic processing with experiment and retrospective analysis (Peters and De Smedt, 2018). It is generally considered that the FPN is in charge of perceiving the top-down activity regulation of the cortex for attention preparation and memory orientation. CON also plays a vital role in the cognitive control, whose downstream effect may be attributed to the output gating of memory. Thus, both the FPN and CON were indispensable in controlling working memory (Wallis et al., 2015). By adopting a functional connectivity analysis, the working memory in the mental arithmetic tasks was also explored and the collaborative work between the frontal lobes and parietal lobes in working memory tasks was detected as well (Hagiwara et al., 2016). However, the DMN was found to be passivated in the mental arithmetic processing, which might be caused by the inhibitory effect of functional network activation during the cognitive tasks (Dimitriadis et al., 2010).

With regards to the big variations in the topological structure of the FPN, it is likely to result from the significant differences in the graph properties between the cognitive states of addition and subtraction in performing the mental arithmetic processing. *Via* the graph analysis and statistical analysis, significant differences can be identified in the adopted metric of the FPN's degree centrality from different brain atlases under the two mental arithmetic cognitive states. Such a result is consistent with the findings of previous studies. For example, the subjects achieved obvious improvements in their mathematics skills and the FPN activities after attending the adaptive number-sense training. It was found that the activation of the subjects' bilateral parietal lobe significantly increased, while the activation of their frontal striatum and middle temporal lobe decreased considerably (Kesler et al., 2011). Moreover, more activation of the FPN is thought to be generated in the numerical inductive reasoning, such as the mental arithmetic process, because more exchanges might be transacted between the intermediate representations and long-term declarative knowledge in the process of numerical rule recognition (Liang et al., 2016). On top of that, the white matter dispersion property of the FPN was also detected to be effective in the prediction of children's mental arithmetic ability (Tsang et al., 2009).

## 5. Conclusion

Focusing on the multi-source cognitive networks, this study takes the single-source cognitive network with the optimal performance as the main cognitive network through synthesizing the multi-source cognitive networks. The ROIs in the supplementary cognitive networks are sorted and integrated into the main cognitive network iteratively, so as to search for the fused cognitive network with the optimal performance in discriminating the graph properties under different cognitive states. The potential advantages of the present research method can be summarized as follows:

1. The distribution of the obtained ROIs in the fused FPN is spatially closer to that of the combined cognitive network, and the ROIs selected are better balanced between the frontal lobe and parietal lobe.
2. The fused cognitive network is constructed under the framework of the main cognitive network by integrating the ROIs with top priority in the supplementary cognitive networks. In the analysis of the fMRI data, the fused cognitive network relies on the main cognitive network for the major interpretation, together with the ROIs of supplementary cognitive networks for the complementary explanation.
3. Compared with other typical machine learning algorithms, the proposed method can yield better performance and the results bear more self-consistency to those obtained in cognitive neuroscience.

On the whole, it has proved that the proposed method can produce a satisfactory evaluation performance and provide a more reasonable interpretation for the related cognitive neuroscience research. However, the potential impact of the fused cognitive network on the cognitive computing model waits for further explorations. Additionally, the generality of such a proposed method also waits for further validations with diverse brain atlases and various fMRI datasets.

## Data Availability Statement

The raw data supporting the conclusions of this article will be made available by the authors, without undue reservation.

## Ethics Statement

The studies involving human participants were reviewed and approved by Xuanwu Hospital of Capital Medical University Ethics Committee. The patients/participants provided their written informed consent to participate in this study.

## Author Contributions

XZ: conceptualization of this study, methodology design and implementation, analysis and interpretation of data, and writing–original draft. YY: cognitive task design and implementation, acquisition of fMRI data, and interpretation of data. HK, JC, and JH: methodology design and implementation. PL: conceptualization of this study and interpretation of data. NZ: conceptualization of this study, methodology design, interpretation of data, and final approval of the version. All authors contributed to the article and approved the submitted version.

## Funding

This work was supported by the National Natural Science Foundation of China (61420106005), the National Key Research and Development Program of China (No. 2020YFB2104402), the JSPS Grants-inAid for Scientific Research of Japan (19K12123), and the Major Projects of Philosophy and Social Science Research in Colleges and Universities in Jiangsu Province, and the Beijing Natural Science Foundation (No. 4222022).

## Conflict of Interest

The authors declare that the research was conducted in the absence of any commercial or financial relationships that could be construed as a potential conflict of interest.

## Publisher's Note

All claims expressed in this article are solely those of the authors and do not necessarily represent those of their affiliated organizations, or those of the publisher, the editors and the reviewers. Any product that may be evaluated in this article, or claim that may be made by its manufacturer, is not guaranteed or endorsed by the publisher.
